# Solid Tranilast Nanocrystal-Loaded Cationic Contact Lenses for Sustained Ocular Drug Delivery

**DOI:** 10.3390/pharmaceutics17101240

**Published:** 2025-09-23

**Authors:** Shinichiro Kobayakawa, Toru Matsunaga, Hiroko Otake, Shiori Hino, Fumihiko Ogata, Manju Misra, Kazutaka Kanai, Naohito Kawasaki, Noriaki Nagai

**Affiliations:** 1 Department of Ophthalmology, Nippon Medical School, Musashi-Kosugi Hospital, 1-396, Kosugi-cho, Nakahara-ku, Kawasaki 211-8533, Kanagawa, Japan; s-kobayakawa@nms.ac.jp; 2 Design and Development, SEED Co., Ltd., 1030-7 Fukuro, Kounosu-shi 369-0131, Saitama, Japan; toru_matsunaga@seed.co.jp (T.M.); s_hino@seed.co.jp (S.H.); 3 Faculty of Pharmacy, Kindai University, 3-4-1 Kowakae, Higashi-Osaka 577-8502, Osaka, Japan; hotake@phar.kindai.ac.jp (H.O.); ogata@phar.kindai.ac.jp (F.O.); kawasaki@phar.kindai.ac.jp (N.K.); 4 Department of Pharmaceutics, National Institute of Pharmaceutical Education and Research, Opposite AirForce Station, Palaj Basan Road, Village Palaj, Gandhinagar 382355, Gujarat, India; manju.misra@lmcp.ac.in; 5 Graduate School of Pharmacy, Gujarat Technological University, Gandhinagar Campus Nr. Government Polytechnic K-6 Circle, E-4 Electronic Estate G.I.D.C, Sector-26, Gandhinagar 382028, Gujarat, India; 6 Department of Small Animal Internal Medicine II, School of Veterinary Medicine, Kitasato University, 35-1 Higashi 23 ban-cho, Towada 034-8628, Aomori, Japan; kanai@vmas.kitasato-u.ac.jp

**Keywords:** tranilast, contact lens, nanocrystal, drug delivery, ophthalmology

## Abstract

**Background/Objectives** Conventional eye drops are the primary therapeutic option for ocular diseases; however, their clinical utility is hindered by several drawbacks, including limited bioavailability and suboptimal patient compliance. To overcome these challenges, we designed a sustained-release contact lens (CL) device loaded with tranilast (TRA) and determined whether the TRA-laden CL could provide sustained drug delivery to the lacrimal fluid and aqueous humor. **Methods** TRA nanocrystals were prepared using the bead-milling approach. Using three types of CLs (nonionic, anionic, and cationic), we prepared TRA-laden CLs by employing a combination of solid TRA nanocrystals and soaking methods under high-temperature and high-pressure conditions in an autoclave (the hThP method). Male Japanese albino rabbits (2–3 kg) were used to evaluate the CLs. **Results** Bead milling reduced the size of the solid TRA nanoparticles (STNs) to approximately 35–180 nm. The TRA-laden cationic CLs prepared using STNs and the hThP method contained a higher amount of TRA than those prepared using the corresponding conventional soaking method. The CLs prepared using the hThP method remained transparent after drug loading. Compared with nonionic and anionic CLs, cationic CLs had the highest drug-loading capacity and allowed for sustained drug release. Moreover, STNs were observed in the released TRA, with no corneal damage or light scattering detected in the rabbits’ eyes. TRA-laden cationic CLs prepared using the hThP method achieved sustained and higher drug delivery into the lacrimal fluid and aqueous humor than those prepared using the conventional soaking method. **Conclusions** Our findings suggest that TRA-laden cationic CLs prepared using STNs and the hThP method can overcome the challenges associated with the conventional soaking method, including low drug uptake and high burst release.

## 1. Introduction

The eye is a highly complex and delicate organ comprising two primary anatomical segments: the anterior and posterior. The anterior segment, located anterior to the lens, comprises the cornea, conjunctiva, iris, lens, ciliary body, and the anterior portion of the sclera. It is further divided into anterior (between the cornea and iris) and posterior (between the iris and lens) chambers, both of which are filled with aqueous humor secreted by the ciliary processes. The aqueous humor plays a pivotal role in nourishing the lens and cornea and maintaining intraocular pressure and undergoes continuous turnover. The structural integrity and functional coordination of these components are essential for visual acuity and overall ocular health [1].

Ocular diseases encompass a wide range of conditions affecting the anterior segment, including cataracts, dry eye, hereditary and degenerative disorders, inflammatory diseases, infectious diseases, glaucoma, tumors, trauma, and ocular manifestations of systemic diseases. Inflammatory ocular diseases involve mast cells, macrophages, and T lymphocytes, which contribute to pathogenesis by releasing cytokines, chemokines, and histamines. These mediators play crucial roles in both physiological and pathological processes. Tranilast (TRA), a cytokine modifier, has demonstrated potential pharmacological benefits in mitigating inflammatory responses.

Ocular drug delivery systems (ODDSs) are primarily used for topical administration (eye drops) [2,3] and injections [4,5,6]. Although conventional eye drops are the primary treatment for anterior segment ocular diseases, their efficacy is limited by ocular barriers, including the cornea, conjunctiva, and tear drainage, allowing only 1–7% of the drug to reach the aqueous humor [7,8]. Although intraocular injections bypass these barriers, this route is invasive and has been associated with poor patient compliance. Moreover, rapid tear turnover and nasolacrimal drainage result in low drug retention and poor drug bioavailability [9,10,11]. Drug permeation is influenced by static barriers (the cornea, tear film, and blood–retinal barrier) and dynamic barriers (tears and conjunctival blood flow). To overcome these challenges, advanced formulation technologies, such as nanomedicines, liposomes, in situ gels, nanocarriers, and drug-eluting devices using contact lenses (CLs) have been explored to enhance ocular bioavailability and treatment efficacy.

Nanotechnology-based drug delivery systems (DDSs) improve therapeutic outcomes by increasing drug absorption and minimizing systemic exposure, offering more effective and sustained ODDSs. These include nanotechnology-based DDSs, such as liposomes, solid lipid nanoparticles (NPs), nanostructured lipid carriers, dendrimers, polymeric NPs, nanoemulsions, dendrimers, polymersomes, inorganic NPs, and protein-based nanomaterials [12,13,14,15,16,17,18]. These innovative systems provide enhanced drug solubility, prolong ocular retention time, improve penetration across the ocular barrier, and facilitate targeted drug delivery to specific ocular tissues. Such advancements contribute to more effective and sustained ocular treatments while minimizing systemic side effects.

In addition, the integration of these technologies has been reported to provide therapeutic effects. Ma et al. reported a novel cyclosporine A nanosuspension (NS@lipid-PEG/CKC) in which nanoscale drug particles are coated with a mixture of lipid DSPE-PEG2000 for mucus penetration and the cationic surfactant cetalkonium chloride (CKC) for enhanced cellular uptake. This combination of PEGylation and cationization suggests a promising strategy for nanomedicine-based eye drops, as it can modulate both mucus penetration and intracellular uptake, thereby improving drug delivery to the ocular epithelium [19]. An example of a nanosuspension formulation targeting ocular surface diseases is Eysuvis, a novel loteprednol etabonate nanosuspension developed by Kala using their AMPPLIFY mucus-penetrating particle (MPP) drug delivery technology. The use of this MPP formulation facilitates the penetration of loteprednol etabonate into target tissues on the ocular surface. Eysuvis is the first ophthalmic corticosteroid approved by the FDA for the treatment of dry eye disease [20]. Integrating NPs with CL devices is a promising strategy for achieving sustained ocular drug delivery. The incorporation of biomolecular aggregates, such as lipophilic vitamins and bovine serum albumin, has been shown to substantially enhance drug encapsulation efficiency in CLs [21,22]. Recent developments in CL-based ODDSs have introduced advanced nanocomposite systems with improved drug loading and controlled release properties [21,22,23]. These systems have shown promise for the effective treatment of ocular diseases with enhanced patient compliance [24,25,26]. In a previous study, we demonstrated that the instillation of dispersions containing solid indomethacin and solid TRA NPs (STNs) increased drug residence time, improved ocular penetration, and enhanced the ocular bioavailability of drugs [27]. In addition, we found that treatment under high-temperature and high-pressure conditions in an autoclave (the hThP method) facilitated drug incorporation into CLs [28]. The hThP method ameliorated problems associated with the conventional soaking method for drug loading in CLs, including a high burst release and low drug uptake [29], which previously limited the application of the soaking method [28]. Additionally, since drug nanoparticles measuring less than 200 nm are smaller than the wavelength of visible light, light scattering is minimal and their impact on vision is expected to be negligible. Thus, we anticipate that by encapsulating drug particles measuring approximately 100 nm within CLs, we can establish a novel device that enables controlled and sustained drug release and absorption. In this study, we designed drug-laden CLs based on a combination of STNs and the hThP method and determined whether the TRA-laden CLs could achieve sustained drug delivery to the lacrimal fluid and aqueous humor. We then developed advanced CLs embedded with STNs by leveraging the intrinsic charge properties of the lenses in combination with the hThP method. Using this approach, we successfully fabricated drug-loaded CLs designed to achieve controlled and sustained release of therapeutic agents. Our experimental results demonstrated that these engineered lenses effectively prolonged the diffusion of the drug into both the lacrimal fluid and aqueous humor. This sustained release profile supports the potential of these CLs as an innovative platform for continuous ocular drug delivery, addressing limitations associated with conventional eye drop administration.

## 2. Materials and Methods

### 2.1. Reagents

Tranilast (TRA) powder and Schirmer’s tear test strips were purchased from Kissei Pharmaceutical Co., Ltd. (Nagano, Japan) and AYUMI Pharmaceutical Co. (Tokyo, Japan), respectively. Nonionic, anionic, and cationic contact lenses (CLs) were provided by SEED Co., Ltd. (Saitama, Japan). 2-hydroxyethyl methacrylate (HEMA) was the primary material in the CLs used in this study. A thermal polymerization initiator was added, and the mixture was heated in an oven to induce copolymerization. The resulting copolymer was then hydrated and swollen to prepare the hydrogel. The raw materials for the three types of CLs (nonionic, anionic, and cationic) were as follows: nonionic, HEMA/ethylene glycol dimethacrylate (EGDMA); anionic, HEMA/EGDMA/1,1,1-trimethylolpropane trimethacrylate/methacrylic acid; and cationic, HEMA/EGDMA/methacryloyl aminopropyl dimethyl benzyl ammonium chloride. All CLs had identical refractive index, diameter, center thickness, and base curve values (1.4 D, 14 mm, 0.065 mm, and 8.7 mm, respectively). In addition, benoxinate hydrochloride (0.4%) and fluorescein (1%) were obtained from Santen Pharmaceutical Co., Ltd. (Osaka, Japan) and Alcon (Tokyo, Japan), respectively. All other chemicals used were of the highest commercially available purity.

### 2.2. Animals

Male Japanese albino rabbits (2–3 kg) were purchased from Shimizu Laboratory Supplies Co., Ltd. (Kyoto, Japan). The animals were housed at 25 °C (light (7:00–19:00)/dark (19:00–7:00)) and allowed free access to commercial feed (CR-3; Clea Japan Inc., Tokyo, Japan) and water. All animal experiments were conducted in accordance with the guidelines for animal research of Animal Research: Reporting of In Vivo Experiments (ARRIVE), the Association for Research in Vision and Ophthalmology (ARVO), and Kindai University. The Animal Experimental Committee of Kindai University approved the experimental protocol on 1 April 2022 (approval no. KAPS-2022-011). The health status and behavior of the rabbits were monitored daily. The rabbits were placed in a holding device during CL application, corneal examination, and sample collection (lacrimal fluid and aqueous humor). Following these procedures, every effort was made to minimize discomfort by immediately releasing the rabbit from the holding device.

### 2.3. High-Performance Liquid Chromatography (HPLC)

The TRA concentration was determined following the methods described in our previous studies, using an Alliance HPLC system (Waters Corporation, Milford, MA, USA) [28]. Briefly, 50 µL of the prepared TRA sample was mixed with 100 µL of an internal standard, ethyl p-hydroxybenzoate (10 µg/mL, dissolved in methanol), while the mobile phase comprised CH_3_CN/50 mM ammonium acetate at a ratio of 20/80 (*v*/*v*, %). An Inertsil ODS-3 column (2.1 × 50 mm, GL Sciences, Tokyo, Japan) was used, and the column temperature was maintained at 35 °C using a CTO-20AC column oven. The flow rate of the mobile phase was set to 0.25 mL/min, with a detection wavelength of 230 nm and a total run time of 11 min. Sample injection was performed using a SIL-20AC autosampler and an injection volume of 10 µL. In this study, a TRA peak was detected between 5 and 7 min.

### 2.4. Preparation of TRA-Laden CLs

Dispersions containing solid TRA micro- and nanoparticles were prepared following the methods described in our previous studies [30,31]. A 0.35% TRA dispersion was prepared by suspending TRA powder in a dispersion medium (pH 7.4) consisting of sodium chloride, a phosphate-buffered solution, Polyoxyethylene(196)Polyoxypropylene(67) Glycol (0.05%), and EDTA. Subsequently, the dispersion was subjected to ultrasonic treatment for 1 h using a W-113 (Honda Electronics, Aichi, Japan). The treated dispersion was then transferred into a 2 mL tube (TOMY, Tokyo, Japan) containing 2 g of zirconia beads (diameter: 0.1 mm), ensuring that the total volume filled approximately 80% of the tube. Bead milling was performed using Micro Smash (5500 rpm, 30 s, 4 °C; Wakenyaku, Kyoto, Japan) alternating with centrifugation (800× *g*, 60 s, 4 °C; TOMY, Tokyo, Japan) for a total of 30 cycles. The resulting dispersion was diluted to 0.15% using a dispersion medium, yielding dispersions containing solid TRA microparticles (STMs) and NPs (STNs). For the experiments, TRA dispersions with or without bead-milling treatment and three types of CLs (nonionic, anionic, and cationic) were utilized. TRA was loaded into the CLs using two methods (soaking and hThP). The soaking method involved incubating the CLs with the TRA dispersions, with or without bead milling, under continuous stirring with a magnetic stirrer for 24 h. The hThP method involved placing the CLs into the TRA dispersions with or without bead milling and treating them under supercritical conditions in an autoclave (pressure: 15 psi; chamber temperature: 121 °C) for 30 min. Thereafter, the CL samples were lightly rinsed with phosphate-buffered saline (PBS) and used in further experiments. To measure the TRA content in the CLs, TRA was extracted by incubating the CLs in 10 mL of PBS for 4 h; this process was repeated 10 times. The TRA concentration in the solution was then determined using the HPLC method. The amount of TRA loaded in the CLs was determined by summing all results of the sample measurements. The osmotic pressure of the TRA suspensions, with or without bead milling, was measured using a micro-osmometer (OM-807; Bio-Medical Science Co., Ltd., Tokyo, Japan).

### 2.5. Measurement of Crystalline TRA

Briefly, the dissolved drug in the dispersions containing STMs and STNs was removed beforehand via high centrifugation (1.0 × 10^5^× *g*) using a Beckman Optima^TM^ MAX-XP Ultracentrifuge (Beckman Coulter, Osaka, Japan) and lyophilized using a freeze dryer (FD-1000; Tokyo Rikakikai Co., Ltd., Tokyo, Japan). The vehicle group was lyophilized without centrifugation. The freeze-drying procedure was performed at −20 °C under a vacuum of 20 Pa for a total duration of 48 h. Powder X-ray diffraction (XRD) and thermogravimetric–differential thermal analysis (TG-DTA) were employed to assess the solid-state properties of the resulting lyophilized powders. XRD patterns were recorded using a Mini Flex II diffractometer (Rigaku Co., Tokyo, Japan) across a 2θ range of 5° to 60° at a scan speed of 10° per minute. The thermal behavior was further characterized using a simultaneous thermal analyzer (DTG-60H; Shimadzu Corp., Kyoto, Japan), in which −5 mg of the sample was analyzed under nitrogen flow. The temperature was increased from 25 °C to 260 °C at a uniform rate of 10 °C/min [30].

### 2.6. Measurement of TRA Particle Size

The TRA characteristics were determined following the methods described in our previous studies [31,32,33]. The drug particle size in dispersions containing STMs and STNs was measured using a SALD-7100 (Shimadzu Corporation, Kyoto, Japan) and a NanoSight LM10 (Malvern, Worcestershire, UK). The measurement conditions for the SALD-7100 were set to maintain the maximum scattered light intensity at approximately 50%, with a refractive index of 1.60 ± 0.10 i. For the NanoSight LM10, the measurement conditions included a viscosity of 1.27 mPa·s, a wavelength of 405 nm, and a measurement duration of 60 s. Scanning electron microscopy (SEM) images were obtained using a NeoScope™ JCM-7000 (JEOL Ltd., Tokyo, Japan).

### 2.7. Drug Release from CLs

Measurements of drug release from the CLs were carried out in accordance with a previous study that evaluated drug release from CLs [28]. PBS (pH 7.4), which is commonly used in experiments involving biological tissues, was selected as the release medium because the CLs evaluated in this study not only release the drug into the tear fluid but also enable its transfer into the eye. Three types of CLs (nonionic, anionic, and cationic) containing TRA were stirred in 4 mL of PBS at 37 °C for 60 h. Aliquots of the PBS were collected at predetermined time points for analysis. For each sample collection time, the CLs were transferred into 4 mL of PBS. The TRA levels in all samples were measured using HPLC. The particle size distribution and particle number in the collected samples were measured using a NanoSight LM10, as described above.

### 2.8. Slit Lamp Examination of CLs in Rabbit Eyes

Rabbits were treated with three types of TRA-containing CLs (nonionic, anionic, and cationic), and the light scattering of the CLs was observed 1 h after application using a slit lamp (METORI-50V; SEED Co., Ltd., Saitama, Japan) equipped with a blue filter. When light scattering occurs within the CL due to drug uptake, the slit lamp illumination is disturbed. Therefore, this method enables the indirect evaluation of changes in the light transmittance of the CL. Observations were performed on five rabbits per group (n = 5).

### 2.9. Corneal Toxicity in Rabbits

The rabbits were treated with TRA-laden cationic CLs for 8 h per day (10:00–18:00) for 7 consecutive days. On day 7 of the experiment, after removing the CLs, the rabbits’ corneas were stained with a solution containing 1% fluorescein and 0.4% benoxinate hydrochloride, and the corneal surface was observed under a METORI-50V slit lamp equipped with a blue filter [28]. Observations were performed on five rabbits per group (n = 5).

### 2.10. Drug Delivery from CLs to Lacrimal Fluid

The drug release from the CLs to the lacrimal fluid was evaluated following the methods described in our previous studies [28]. Briefly, rabbits were treated with TRA-laden cationic CLs, and lacrimal fluid samples were collected using Schirmer’s tear test strips at 0.5, 1, 2, 4, and 6 h after CL application. The Schirmer’s tear test strips, including the absorbed samples, were homogenized in methanol to extract TRA. The TRA concentration was determined using the HPLC method. We also used the trapezoidal method to calculate the area under the TRA concentration–time curve (*AUC*_0–6h_) up to a final measurement time of 6 h.

### 2.11. Drug Delivery from CLs to Aqueous Humor

Drug delivery from the CLs to the aqueous humor was measured following the method for the ocular drug penetration of eye drops described in a previous study [27,32,33,34]. Briefly, the rabbits were anesthetized using isoflurane, and a topical anesthetic (0.4% benoxinate hydrochloride) was administered to each eye 3 min prior to aqueous humor sampling. A 29 G injection needle, connected to silicone tubing (inner diameter: 0.5 mm; Fuji Systems Co., Tokyo, Japan) and attached to a 25 µL microsyringe (Ito Corp., Tokyo, Japan), was inserted into the eye and left in place for 30 min to allow stabilization. After the stabilization period, the rabbits were administered TRA-laden cationic CLs. Aqueous humor samples (5 µL each) were periodically collected from the anterior chamber at 5, 10, 20, 30, 40, 50, 60, 70, 80, and 90 min after CL application. The TRA concentrations were quantified using HPLC. Additionally, the area under the TRA concentration–time curve (*AUC*_0–90min_), up to a final measurement time of 90 min, was calculated using the trapezoidal method.

### 2.12. Inhibitor of Energy-Dependent Endocytosis

Endocytosis was inhibited using the method reported previously, employing three types of inhibitors [34]. Caveolae-dependent endocytosis was inhibited with 54 µM nystatin, which acts by binding to plasma membrane cholesterol. Clathrin-dependent endocytosis was inhibited with 40 µM dynasore, a specific and highly effective blocker of dynamin, one of the key proteins in the endocytosis machinery of synaptic vesicles. Micropinocytosis was inhibited with 2 µM of rottlerin, a selective inhibitor of fluid-phase endocytosis. All endocytosis inhibitors were dissolved in 0.5% DMSO and instilled 10 min prior to the application of TRA-laden cationic CLs prepared using STNs and the hThP method.

### 2.13. Statistical Analysis

Data analyses were performed using JMP version 5.1 (SAS Institute, Cary, NC, USA). The data are presented as means ± standard errors (SEs). Comparisons between groups were performed using Student’s *t*-test and analysis of variance (ANOVA), followed by the Tukey–Kramer test. A *p*-value of <0.05 was considered statistically significant.

## 3. Results

### 3.1. STN Loading in CLs Using the hThP Method

**Figure 1:**
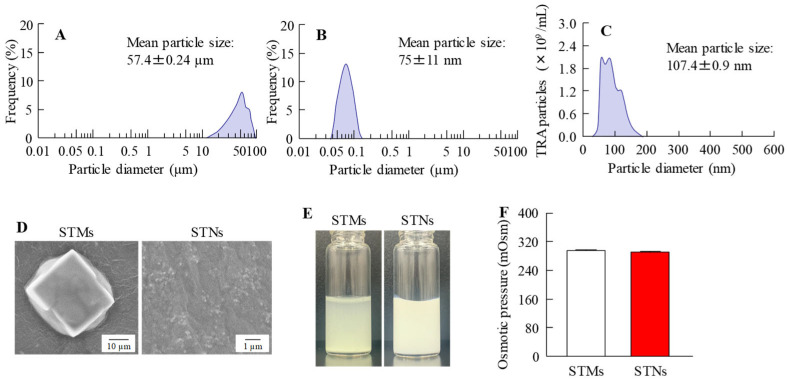
Drug size and osmotic pressure of TRA dispersions used during drug loading into CLs. (**A**,**B**) Particle size frequencies of TRA suspensions with (STNs) or without (STMs) bead milling, as determined using SALD-7100. (**C**) Particle size frequencies of STNs, as determined using Nanosight LM10. (**D**–**F**) SEM (**D**) and digital (**E**) images and osmotic pressure (**F**) of TRA suspensions with (STNs) and without (STMs) bead milling. The particle size of TRA was reduced to approximately 35–180 nm upon bead milling.

**Figure 2:**
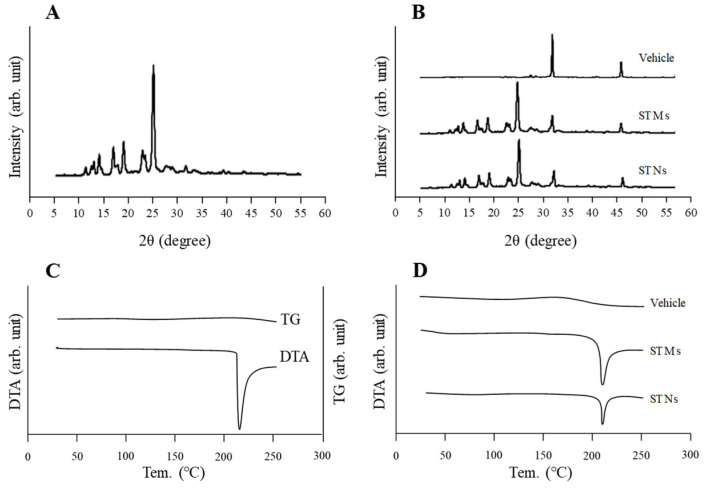
XRD and TG-DTA patterns of TRA with or without bead-milling treatment. (**A**) XRD pattern of TRA powder. (**B**) Changes in XRD patterns of vehicle and TRA with or without bead milling. (**C**) TG and DTA patterns of TRA powder. (**D**) Changes in DTA patterns of vehicle and TRA with and without bead milling. No differences in XRD and DTA patterns can be observed between TRA with and without bead-milling treatment.

**Figure 3:**
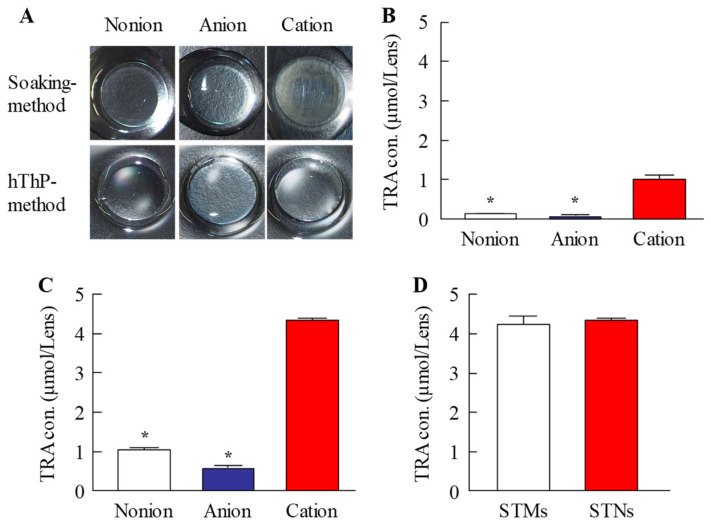
Amount of TRA loaded into CLs using the soaking and hThP methods. (**A**) Digital images of nonionic, anionic, and cationic CLs prepared with solid TRA nanoparticles (STNs) and subjected to soaking or the hThP method. (**B**,**C**) Amount of TRA in nonionic, anionic, and cationic CLs loaded with STNs and subjected to soaking (**B**) or the hThP (**C**) method. (**D**) Amount of TRA in cationic CLs loaded with solid TRA microparticles (STMs) and STNs using the hThP method. n = 5. * *p* < 0.05 vs. cationic CLs for each category. TRA-laden cationic CLs prepared using STNs and the hThP method possess more TRA than TRA-laden cationic CLs prepared using STNs and the soaking method. In contrast, TRA levels in cationic CLs loaded with STNs using the hThP method are similar to those in cationic CLs loaded with STMs using the hThP method. CLs subjected to the hThP method remain transparent after drug loading. Cationic CLs exhibit the highest drug-loading capacity.

To incorporate nanocrystals into CLs, we first prepared a dispersion containing nanosized TRA using a wet bead-milling process. Figure 1A–D present the particle size distributions of the TRA before and after bead milling. The bead-milling process successfully reduced the TRA particle size from 57.4 µm to 75 nm, achieving colloidal-level refinement (Figure 1A–C). In addition, the prepared nanosized TRA dispersion exhibited a more uniform color compared with the microsized suspension, appearing closer to white (Figure 1E). Furthermore, SEM images confirmed that large aggregates of TRA were successfully reduced to fine particles (Figure 1D). The osmotic pressure in the TRA dispersions with and without bead-milling treatment was maintained at similar levels of 296.2 and 290.5 mOsm/L, respectively (Figure 1F). Since TRA was successfully reduced to the nanoscale, we examined how its crystal structure was affected by this process (Figure 2). The results showed that the XRD and TG-DTA patterns of the TRA with and without bead-milling treatments were similar. In addition, the peak levels of TG-DTA in the TRA dispersions prepared with (peak temperature 209.8 °C) and without (peak temperature 209.5 °C) autoclaving (pressure: 15 psi; chamber temperature: 121 °C, 30 min) were similar. These findings indicated that the crystalline structure was preserved even after bead milling and autoclave treatment. Subsequently, we examined whether the prepared STN could be loaded onto three different types of CLs. Figure 3A presents images of the CLs obtained upon TRA loading using a combination of STNs and the hThP method. The CLs processed using the hThP method—including nonionic, anionic, and cationic CLs—remained transparent, with no visible degradation. This study used CLs made with HEMA hydrogels, and autoclaving is a standard procedure for sterilizing these CLs. Therefore, this process did not affect the refractive index (1.4 D), diameter (14 mm), center thickness (0.065 mm), or base curve (8.7 mm) of any of the CLs processed using the hThP method. These results suggest that autoclaving may not affect the mechanical properties of these CLs. We also quantified the amount of TRA loaded onto the CLs using the hThP method (Figure 3B,C). The TRA content in the cationic CLs was 2.5- and 4.7-fold higher than in the nonionic and anionic CLs, respectively. The TRA content in the CLs prepared using STNs and the hThP method was similar to that observed in the CLs prepared using STM (without bead milling) and the hThP method (Figure 3D).

### 3.2. Evaluation of Drug Behavior and Release from STN-Laden CLs

**Figure 4:**
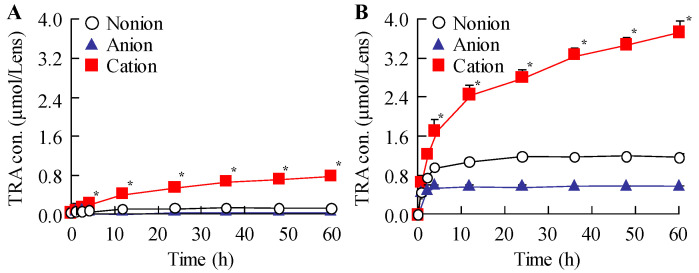
Drug-release behavior from TRA-laden CLs. Changes in release profiles of nonionic, anionic, and cationic CLs subjected to soaking (**A**) or the hThP method (**B**) (n = 5). * *p* < 0.05 vs. cationic CLs for each category. Drug-laden cationic CLs achieve sustained and high drug release from CLs.

**Figure 5:**
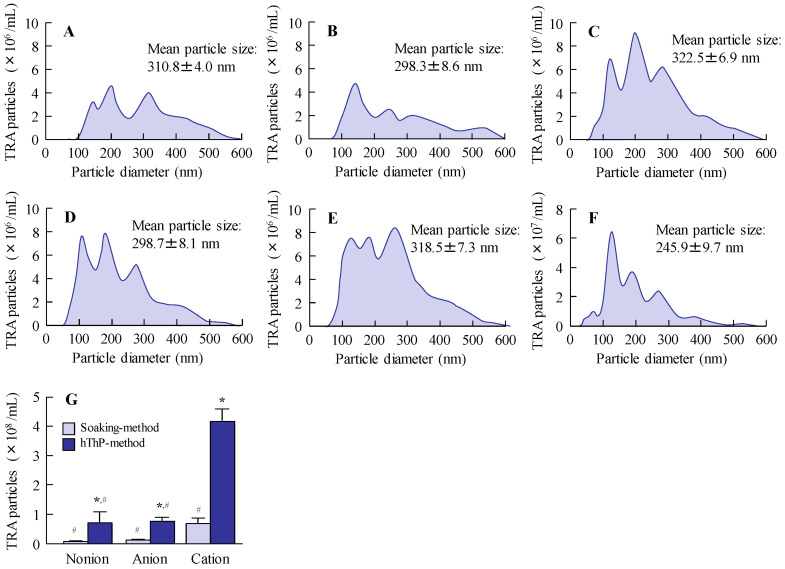
Particle size and particle count of drug released from TRA-laden CLs using solid TRA nanoparticles (STNs). (**A**–**C**) Particle size frequencies of TRA released from nonionic (**A**), anionic (**B**), and cationic (**C**) CLs subjected to the soaking method. (**D**–**F**) Particle size frequencies of TRA released from nonionic (**D**), anionic (**E**), and cationic CLs (**F**) subjected to the hThP method. (**G**) Number of TRA NPs released from nonionic, anionic, and cationic CLs subjected to soaking and hThP methods, respectively (n = 5). * *p* < 0.05 vs. soaking method for each category. ^#^ *p* < 0.05 vs. cationic CLs using the soaking method for each category. TRA appears to be released as NPs in all CLs loaded with STNs. Cationic CLs released the highest number of TRA NPs.

After confirming that TRA could be incorporated into the CLs, we evaluated the drug behavior and release profile in the STN-loaded CLs. Figure 4 illustrates the results of the drug-release assessment for TRA-laden CLs prepared using STNs and the hThP method. In the nonionic and anionic CLs, TRA release plateaued at 10 and 1 h after initiating the experiment, respectively. Conversely, TRA release from the cationic CLs persisted for up to 60 h. After 24 h, the release of TRA from the cationic CLs was approximately 4.1- and 7.8-fold higher than its release from nonionic and anionic CLs, respectively, indicating that the cationic CLs achieved the highest TRA release among the CLs examined. The particle size and amount of TRA released from the CLs are shown in Figure 5. TRA was released in NP form from all CLs, that is, nonionic, anionic, and cationic CLs, with particle sizes in the 50–600 nm range (Figure 5A–F). In addition, the highest number of TRA NPs was released from the TRA-laden cationic CLs prepared using STMs and the hThP method (Figure 5G). In this way, the TRA-loaded cationic CLs prepared using STM and the hThP method were shown to be the most optimal in terms of TRA incorporation and release.

### 3.3. Changes in TRA Levels in the Lacrimal Fluid and Aqueous Humor of Rabbits Treated with STN-Laden Cationic CLs

**Figure 6:**
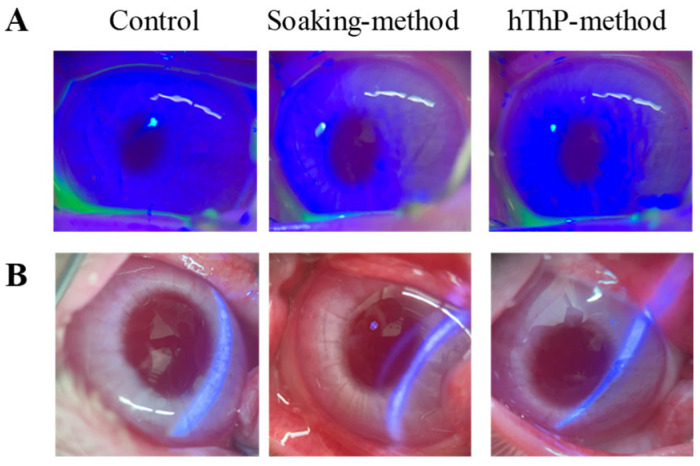
Changes in the cornea and light scattering in the rabbit eye upon application of TRA-laden cationic CLs prepared using STNs and the hThP method. (**A**) Representative images of rabbit corneas after CL application. CLs were repeatedly applied to the rabbit eye for 8 h per day for 7 days. (**B**) Slit lamp image of CL in a rabbit eye treated with blue light. No corneal damage or light scattering can be observed in the rabbit eye, even after application of TRA-laden CLs prepared using STNs and the hThP method.

**Figure 7:**
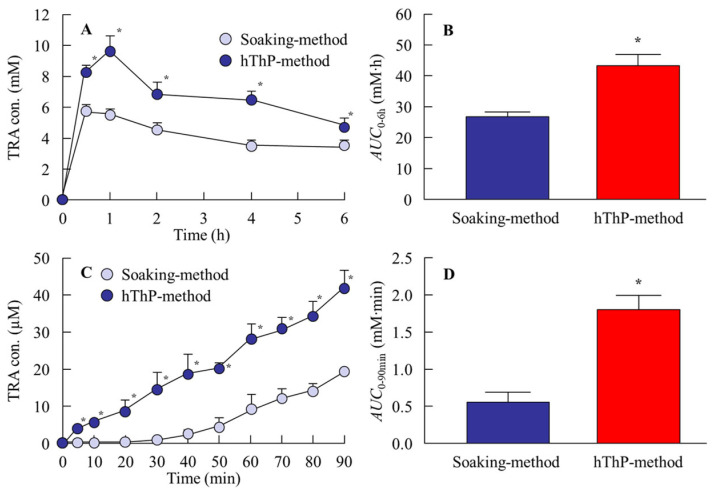
TRA behavior in lacrimal fluid and aqueous humor upon application of TRA-laden cationic CLs prepared using the soaking and hThP methods. (**A**,**B**) Profile (**A**) and *AUC*_0–6h_ (**B**) in lacrimal fluid of rabbits treated with TRA-laden CLs. (**C**,**D**) Profile (**C**) and *AUC*_0–90min_ (**D**) in aqueous humor of rabbits treated with TRA-laden CLs. n = 6. * *p* < 0.05 vs. soaking method for each category. TRA-laden cationic CLs prepared using the hThP method exhibit sustained and higher drug release into the lacrimal fluid than those prepared using the soaking method. Application of TRA-laden CLs enables sustained drug delivery into the aqueous humor. The ocular permeability of TRA from CLs subjected to the hThP method is higher than that from CLs subjected to the soaking method.

**Figure 8:**
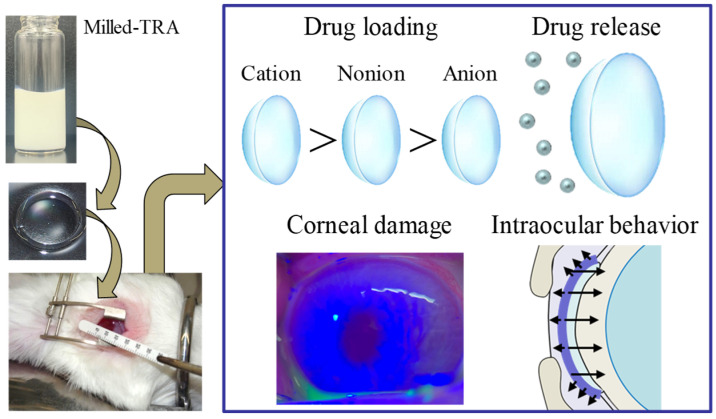
Schematic diagram of TRA-laden CLs illustrating drug incorporation, release, corneal damage, and intraocular behavior.

It is crucial to examine and establish the safety, drug-release properties, and behavior of TRA-laden CLs. In this study, we determined the ocular stimulation of rabbits treated with TRA-laden cationic CLs (Figure 6A). Examination for corneal damage after continuous treatment with TRA-laden cationic CLs (cationic CLs subjected to the soaking and hThP methods) for 8 h per day over 7 days revealed no corneal injury, similar to the non-drug-laden CLs (control). Additionally, there were no signs of conjunctival hyperemia or discomfort-related behaviors during CL application, and no light scattering was detected even after applying TRA-laden cationic CLs prepared using STNs and the hThP method (Figure 6B). Figure 7 illustrates the behavior of TRA in the lacrimal fluid (Figure 7A) and aqueous humor (Figure 7B) following the application of the TRA-laden cationic CLs. TRA-laden cationic CLs prepared using both the soaking and hThP methods released TRA into the lacrimal fluid and aqueous humor. The level of TRA released from the TRA-laden cationic CLs prepared using the hThP method was significantly higher than that released from the TRA-laden cationic CLs prepared using the soaking method. The *AUC* (*AUC*_0–6h_ and *AUC*_0–90min_) levels of the CLs subjected to the hThP method were 1.62- and 3.23-fold greater than those of the CLs subjected to the soaking method, respectively. In this study, we investigated how ocular drug distribution changed upon pretreatment with three endocytosis inhibitors, including nystatin (a caveolae-dependent endocytosis inhibitor), dynasore (a clathrin-dependent endocytosis inhibitor), and rottlerin (a micropinocytosis inhibitor). The *AUC*_0–90min_ in the aqueous humor of rabbits treated with TRA-laden cationic CLs prepared using STNs and the hThP method was 1.1 mM∙min (n = 3), and corneal penetration was prevented via pretreatment with endocytosis inhibitors. These results demonstrate that the TRA-laden cationic CLs prepared using the hThP method exhibited higher drug loading and sustained release compared with the other CL materials used in this study. Furthermore, they showed no significant impact on corneal integrity or visual field. Our findings also indicate that endocytosis is a major pathway for drug transfer into the aqueous humor (Figure 8).

## 4. Discussion

Conventional eye drops are the primary therapeutic option for ocular diseases. Nevertheless, their clinical applicability is hindered by drawbacks such as limited bioavailability, potential adverse reactions, and suboptimal patient compliance [35]. To overcome these challenges, researchers have explored the integration of two distinct, innovative technologies, including nanotechnology, which have been reported to yield therapeutic effects. Ma et al. reported a novel cyclosporine A nanosuspension (NS@lipid-PEG/CKC) in which nanoscale drug particles are coated with a mixture of lipid DSPE-PEG2000 for mucus penetration and the cationic surfactant CKC for enhanced cellular uptake [19]. A nanosuspension of Eysuvis, a novel loteprednol etabonate nanosuspension, was developed to target ocular surface diseases [20]. Integrating NPs with CL devices is also considered a promising strategy for achieving sustained ocular drug delivery. The incorporation of biomolecular aggregates, such as lipophilic vitamins and bovine serum albumin, has been shown to substantially enhance drug encapsulation efficiency in CLs [21,22]. Recent developments in CL-based ODDSs have introduced advanced nanocomposite systems with improved drug loading and controlled release properties [21,22,23]. Researchers have explored drug-releasing CLs as an alternative approach to improve intraocular drug delivery, and integrating NPs into CLs has emerged as a promising strategy for prolonging drug release and enhancing therapeutic outcomes [36,37]. These advanced delivery platforms have also demonstrated considerable potential to optimize ocular disease management while improving patient adherence [24,25,26]. In a previous study, we showed that administering dispersions containing solid nanocrystals could effectively extend the drug retention period, enhance corneal permeability, and improve drug bioavailability [27]. Furthermore, our study revealed that the hThP method successfully addressed the critical limitations of the conventional soaking technique for loading drugs into CLs—including excessive burst release and inadequate drug uptake—which previously restricted their applicability [28]. In this study, we engineered CLs embedded with STNs using the differently charged CL and hThP methods. Our results confirm that these drug-laden CLs facilitated prolonged drug diffusion into the lacrimal fluid and aqueous humor, supporting sustained ocular drug delivery (Figure 8).

We first prepared STNs and investigated whether the combined use of STNs and the hThP method improved drug uptake. In our previous study, we found that TRA could be reduced to the nanoscale using a breakdown method, specifically, bead-milling treatment. In this study, we prepared STNs as described previously [31,33]. The results indicated that the TRA particles processed via wet bead milling had a size range of 35–180 nm (Figure 1). Additionally, XRD and TG-DTA confirmed that the crystal structure of TRA was unchanged by bead milling (Figure 2). Therefore, we attempted to incorporate these STNs into CLs. The most widely used method of loading ocular drugs into CLs is the soaking method [29]. However, the soaking method is associated with issues such as high burst release and low drug uptake [29]. We found that we were able to overcome these issues by preparing TRA-laden CLs using the hThP method. Accordingly, we aimed to prepare drug-laden CLs with STNs and the hThP method [28], using CLs with different charges (nonionic, anionic, and cationic). In all three types of CLs, the TRA content in CLs subjected to the hThP method was higher than that in CLs subjected to the conventional soaking method (Figure 3). The TRA content in drug-laden cationic CLs prepared using STNs and the hThP method was similar to that in drug-laden cationic CLs prepared using STMs (without bead milling) and the hThP method (Figure 3D). Typically, particle movement and solubility in solvents increase under hThP, which may lead to higher incorporation of TRA into CLs. The cationic CLs exhibited the highest drug-loading capacity among all three types of CLs, regardless of whether they were soaked or subjected to the hThP method (Figure 3). The three types of CLs used in this study possess distinct ion charges, while TRA is a weakly acidic drug containing carboxylic acid. Carboxylic acids dissociate under neutral conditions, forming a negative charge (R-COO^−^). Therefore, it was hypothesized that COO^−^ (carboxylic acids with a negative charge) can bind more easily to the cationic part of cationic CLs than to nonionic and anionic CLs under neutral conditions. Thus, the difference in ionic charge may impact the content and release of TRA from these CLs. We also examined the effect of TRA loading on the visual field. For all three types of CLs, the soaking and hThP methods both produced transparent CLs (Figure 3A). Moreover, no light scattering was observed upon applying drug-laden CLs prepared using STNs and the hThP method (Figure 6B). These results suggest that the application of TRA-laden CLs prepared using STNs may not affect the visual field.

Subsequently, we examined the drug release behavior of the loaded CLs. The particle size distribution was measured to determine whether TRA was released as solid particles or in a dissolved state. All drug-laden CLs prepared using STNs and the hThP method exhibited greater TRA release than drug-laden CLs prepared using the soaking method (Figure 4). Moreover, TRA-laden cationic CLs showed higher drug release than TRA-laden nonionic and anionic CLs prepared using the soaking and hThP methods, respectively (Figure 4). We observed the release of TRA NPs from all CLs used in this study, with the cationic CLs releasing the highest number of TRA NPs (Figure 5). Drug loading and release behaviors are known to be influenced by the CL composition [38,39] such that low and high affinities can result in either rapid release or no release, respectively. The water content and ionic charge have also been reported to play crucial roles in determining the affinity between the CL and the drug [38,39,40]. In this study, the water contents of the three types of CLs used—nonionic, anionic, and cationic—were 38%, 58%, and 46%, respectively. However, the differences in water content between these CLs did not correlate with their drug-release behavior, as the highest drug loading and release were demonstrated by the cationic CLs, which had a moderate water content among the three types of CLs used in this study. Therefore, similar to the enhanced incorporation of TRA into the cationic CLs due to the inclusion of carboxylic acids, differences in ionic charge may influence drug release from CLs. In this study, we used CLs manufactured with materials and equipment that are already in practical use. Furthermore, the drug-loaded cationic CLs were evenly divided into eight segments, and the drug content of each segment was measured. No significant variation was observed (4.31 ± 0.04 µmol/segment (mean ± SD); n = 20). In addition, no light scattering was observed in any part of the CLs after drug loading (Figure 6). This uniform drug encapsulation suggests that the drug was evenly distributed throughout the entire CL. Further detailed evaluation of the characteristics of the CLs may help to refine strategies for blocking transmittance. Thus, further investigations are required; we plan to investigate the encapsulation of drugs with different charges and to examine how sustained-release properties change when the CL material is altered to explore the relationships between the material and drug loading, release kinetics, and nanoparticle behavior in greater detail.

It is essential to clarify both the safety and ocular pharmacokinetics of the drug for practical applications. Therefore, we examined potential corneal toxicity during CL application (Figure 6) and drug delivery from the CLs into the lacrimal fluid and aqueous humor (Figure 7). The safety of TRA-laden cationic CLs prepared using STNs and the soaking and hThP methods was evaluated in rabbit eyes. Corneal damage was assessed after the repeated application of drug-laden CLs for 8 h per day over 7 days, and no corneal injury was observed in either group (Figure 6A). Additionally, no signs of conjunctival hyperemia or pain-related behavior were observed during CL application. These findings indicate that the designed CLs are safe for repeated use. Subsequently, the ophthalmic behavior of TRA was determined after the application of TRA-laden cationic CLs prepared using STNs and the soaking and hThP methods (Figure 7). Herein, TRA-laden cationic CLs prepared using STNs and the hThP method achieved more sustained and higher drug release into the lacrimal fluid than those prepared using the soaking method (Figure 7A). Furthermore, the use of TRA-laden cationic CLs facilitated drug permeation through ocular tissues such as the cornea, allowing the drug to reach the aqueous humor (Figure 7B). The TRA-laden CLs prepared using the hThP method facilitated more sustained drug delivery into the aqueous humor and higher concentrations than CLs prepared using the soaking method. The high tissue permeability of NPs is generally mediated by endocytotic mechanisms [41,42,43,44]. We previously demonstrated that the instillation of drug dispersions containing NPs can achieve higher corneal permeability than the instillation of dispersions comprising solid microparticles, with endocytosis playing a role in the permeation process within ocular tissues [34]. In this study, TRA was released from the CLs in the form of NPs (Figure 5). In addition, high corneal penetration was inhibited via pretreatment with endocytosis inhibitors. These results suggest that the use of NPs contributes to enhanced tissue permeability. Nevertheless, additional studies are needed to elucidate the therapeutic effects of the prepared TRA-laden cationic CLs on allergic conjunctivitis, which we plan to conduct in the future. Notably, TRA, which was used in this study, is an anti-allergic agent already in clinical use as an ophthalmic solution, and its efficacy has been demonstrated when the drug concentration in the tear fluid reaches a certain level. In this study, we measured the concentrations of TRA in the tear fluid and aqueous humor over time and confirmed that the levels likely to exert a pharmacological effect were maintained (Figure 7). Therefore, even without the use of experimental animal models, the formulation’s utility can be evaluated, and these drug-loaded cationic CLs are expected to exhibit sufficient pharmacological efficacy. However, to ensure rigor and reproducibility, we consider it essential to further optimize ocular evaluation protocols in animal models and to incorporate cell-based assays to strengthen the mechanistic evidence. Collectively, our findings suggest that the use of cationic CLs loaded with the anti-allergic drug TRA could achieve prolonged suppression of inflammation both on the ocular surface and within intraocular tissues. These findings suggest that our approach offers a noninvasive alternative to existing commercially available extended-wear ophthalmic systems, such as drug-eluting intraocular lens inserts. Furthermore, compared with conventional drug-loaded CLs, our approach has the potential to enhance the drug-loading capacity and prolong the drug release duration. Moreover, it can reduce the need for frequent instillation, such as with eye drops. In addition, when used in combination with devices such as punctal plugs, it may provide greater therapeutic efficacy than conventional methods.

## 5. Conclusions

The findings of this study revealed that TRA-laden cationic CLs prepared using STNs and the hThP method exhibited more effective TRA uptake than both drug-laden CLs prepared using the conventional soaking method and those prepared using STMs and the hThP method. Moreover, the TRA-laden cationic CLs prepared using STNs and the hThP method remained transparent, exhibited no corneal damage upon application, and showed no signs of light scattering. Upon applying the TRA-laden cationic CLs prepared using STNs and the hThP method, the drug was released in the form of NPs, enabling sustained drug delivery to both the lacrimal fluid and aqueous humor. Taken together, these findings suggest that the TRA-laden cationic CLs prepared using STNs and the hThP method could overcome the limitations associated with the conventional soaking method, including low drug uptake and high burst release (Figure 8). TRA is an anti-allergic drug that has been clinically applied as an ophthalmic solution, and its efficacy when the concentration in the tear fluid reaches a certain level has already been proven. Therefore, this study focused primarily on changes in drug concentration. Evaluating the drug’s efficacy in models of inflammation or allergic conjunctivitis will be important in future studies. We plan to expand our research to cover potential clinical translation challenges, such as the manufacturing scalability of the nanocrystal-loaded lenses, their long-term comfort and wearability, and the implications for patient adherence.

## Data Availability

The raw data supporting the conclusions of this article will be made available by the authors upon request.

## Abbreviations

*AUC*	Area under the drug concentration–time curve
CKC	Cetalkonium chloride
COO^−^	Carboxylic acid
CL	Contact lens
EGDMA	Ethylene glycol dimethacrylate
HEMA	2-hydroxyethyl methacrylate
hThP method	Soaking method under high temperature and high pressure
MPP	Mucus-penetrating particle
NP	Nanoparticle
ODDS	Ocular drug delivery system
SE	Standard error
SEM	Scanning electron microscopy
STMs	Solid tranilast microparticles
STNs	Solid tranilast nanoparticles
TG-DTA	Thermogravimetry-differential thermal analysis
TRA	Tranilast
XRD	X-ray diffraction
